# Glomerular Endothelial Cell Injury and Damage Precedes That of Podocytes in Adriamycin-Induced Nephropathy

**DOI:** 10.1371/journal.pone.0055027

**Published:** 2013-01-24

**Authors:** Yu Bo Yang Sun, Xinli Qu, Xueming Zhang, Georgina Caruana, John F. Bertram, Jinhua Li

**Affiliations:** 1 Department of Anatomy and Developmental Biology, Monash University, Wellington Road, Clayton, Victoria, Australia; 2 Baotou Medical College, Inner Mongolia University of Science and Technology, Baotou, People's Republic of China; Fondazione IRCCS Ospedale Maggiore Policlinico & Fondazione D'Amico per la Ricerca sulle Malattie Renali, Italy

## Abstract

The role of podocytes in the development and progression of glomerular disease has been extensively investigated in the past decade. However, the importance of glomerular endothelial cells in the pathogenesis of proteinuria and glomerulosclerosis has been largely ignored. Recent studies have demonstrated that endothelial nitric oxide synthatase (eNOS) deficiency exacerbates renal injury in anti-GBM and remnant kidney models and accelerates diabetic kidney damage. Increasing evidence also demonstrates the importance of the glomerular endothelium in preventing proteinuria. We hypothesize that endothelial dysfunction can initiate and promote the development and progression of glomerulopathy. Administration of adriamycin (ADR) to C57BL/6 mice, normally an ADR resistant strain, with an eNOS deficiency induced overt proteinuria, severe glomerulosclerosis, interstitial fibrosis and inflammation. We also examined glomerular endothelial cell and podocyte injury in ADR-induced nephropathy in Balb/c mice, an ADR susceptible strain, by immunostaining, TUNEL and Western blotting. Interestingly, down-regulation of eNOS and the appearance of apoptotic glomerular endothelial cells occurred as early as 24 hours after ADR injection, whilst synaptopodin, a functional podocyte marker, was reduced 7 days after ADR injection and coincided with a significant increase in the number of apoptotic podocytes. Furthermore, conditioned media from mouse microvascular endothelial cells over-expressing GFP-eNOS protected podocytes from TNF-α-induced loss of synaptopodin. In conclusion, our study demonstrated that endothelial dysfunction and damage precedes podocyte injury in ADR-induced nephropathy. Glomerular endothelial cells may protect podocytes from inflammatory insult. Understanding the role of glomerular endothelial dysfunction in the development of kidney disease will facilitate in the design of novel strategies to treat kidney disease.

## Introduction

Diabetic and non-diabetic glomerular diseases remain the major cause of chronic and end-stage renal disease [1]. Proteinuria is an indicator of kidney disease and is largely caused by glomerular disease, such as diabetes or glomerulonephritis [2]. Numerous studies have focused on the roles of podocytes, the glomerular basement membrane (GBM) and mesangial cells in the pathogenesis of proteinuria and glomerulosclerosis [3]–[5]. The importance of glomerular endothelial cells in glomerular injury has been largely ignored. Recent studies have demonstrated that endothelial nitric oxide synthatase (eNOS) deficiency exacerbates renal injury in anti-GBM [6] and remnant kidney models [7] and accelerates diabetic kidney damage with features that resemble human diabetic nephropathy (DN) [8]–[11]. In patients, *eNOS* polymorphisms that lead to decreased eNOS expression and activity have been associated with advanced DN and progressive IgA nephropathy [12]–[14]. Scavengers of endothelial nitric oxide (NO)-production, such as asymmetric dimethyl-arginine or N-Nitro-L-Arginine Methyl Ester (L-NAME) can acutely increase glomerular permeability and induce proteinuria [15]–[17]. Collectively, these studies suggest that endothelial dysfunction is involved in the development of diabetic and non-diabetic glomerular injury and renal fibrosis [18], [19].

One of the most important mediators released by the endothelium is NO. NO acts as a potent vasodilator, and also inhibits inflammation, growth of vascular smooth muscle and aggregation of platelets [20]–[23]. Dysregulation of NO has been described in patients with DN, including increased NO expression in early DN, followed by marked down-regulation. Henke et al. [24] generated mice in which the nuclear factor kappa B (NF-κB) suppressor IκBαΔ was induced in the endothelium using Cre/Lox technology. When these mice were exposed to Angiotensin II infusion, high salt and inhibition of endogenous NO production, hypertension was not prevented. However, NF-κB suppression markedly reduced renal injury as evidenced by decreased proteinuria, renal inflammation and fibrosis [24]. This study demonstrated a previously unappreciated role of the endothelium in glomerular injury [25].

It is believed that the glomerular filtration barrier (GFB), including the podocyte layer, the glomerular basement membrane (GBM), and the endothelium, plays an essential role in regulating glomerular permeability. Recent studies have demonstrated the importance of the glomerular endothelium and its surface layer in preventing proteinuria [26]. Increasing evidence also demonstrates that glomerular endothelial cell fenestrae are integral components of the glomerular filtration barrier [27]–[31]. Reduction in glomerular endothelial cell fenestration and an increase in podocyte detachment are correlated with the severity of classical DN lesions and renal function in type 1 diabetic patients [32]. Taken together, these studies from both structural and functional viewpoints demonstrate that glomerular endothelial dysfunction plays a critical role in the pathogenesis of progressive renal disease, suggesting that endothelial function is also a key determinant of susceptibility to nephropathy.

In the present study we hypothesize that endothelial dysfunction can initiate and propel the development and progression of glomerulopathy. We tested whether eNOS deficiency promotes endothelial injury and drives the development of adriamycin (ADR)-induced nephropathy in C57BL/6 mice, an ADR-resistant strain. We also examined and compared podocyte and glomerular endothelial cell injury in ADR-induced nephropathy in Balb/c mice, an ADR-susceptible strain. Finally we investigated whether the conditioned medium from mouse microvascular endothelial cells over expressing eNOS can protect podocytes from TNF-α-induced injury *in vitro*.

## Materials and Methods

### Experimental Animals

Wild-type C57BL6/J and Balb/c mice (8 weeks old) were purchased from Monash Animal Services, Monash University, Australia. Breeding pairs of *eNOS* knockout mice were purchased from Jackson Laboratories (Bar Harbor, ME) and maintained at Monash Animal Services. All experiments were performed with the approval of a Monash University Animal Ethics Committee, which adheres to the “Australian Code of Practice for the Care and Use of Animals for Scientific Purposes.” Five C57BL/6 male mice and six Balb/c male mice per group were used in each experiment.

To establish the animal model of adriamycin (ADR)-induced nephropathy, wild type, *eNOS* knockout and Balb/c mice received a single intravenous injection of ADR (10.5 mg/kg; Sigma, St. Louis, MO). Control mice were treated with an equivalent intravenous volume of normal saline (NS) vehicle. Mice were killed at 24 hours, 72 hours, 1 week, 2 weeks, and 4 weeks after ADR or NS injection. Cardiac blood, urine and kidney tissue were collected for analysis. Six mice per group were used in these studies.

### Blood Pressure Measurements

Systolic blood pressure (BP) was measured using a tail-cuff sphygmomanometer (Visitech BP2000; Visitech Systems, Apex, NC). Animals were trained and accustomed to the machine, and all measurements were performed at the same time of day.

### Measurements of creatinine and protein in blood and urine

Mice were housed in metabolic cages, with free access to chow and water on the days of urine collection. Protein from 24 hour urine samples and serum creatinine levels were measured using a *DC Protein Assay kit* (Bio-Rad, Gladesville, New South Wales, Australia) and Creatinine Assay kit (Cayman Chemical, Ann Abor, MI), according to instructions supplied.

### Cell Culture

#### Mouse podocyte cell culture

Podocytes between passage 10 and 15 were maintained in RPMI 1640 medium supplement with 10% fetal bovine serum (FBS) and 1% streptomycin/penicillin solution [33]. Cells were propagated in 10 U/ml murine IFNγ at 33°C and then differentiated by culture for 7 days at 37°C in the absence of IFNγ [34]. Differentiated podocytes showed prominent cytoplasmic processes and expressed synaptopodin.

#### Mouse microvascular endothelial cell (MMEC) culture and generation of eNOS over-expression MMECs

MMECs were purchased from ATCC (Manassas, VA ) and cultured in 5% CO_2_ atmosphere at 37°C in Dulbecco's modified Eagle's medium (Life Technologies BRL, Gaithersburg, MD) containing 10% FBS. To generate eNOS over-expression in MMECs, MMECs were transfected with pcDNA3-eNOS-GFP plasmid (Addgene Plasmid 22444) using FuGENE HD (Roche, Hawthorn, Austrialia). Seven days after transfection, two rounds of fluorescence activated cell sorting (FACS) (FACsDiva, Flowcore, Clayton, Australia) were employed to obtain eNOS-GFP-positive and eNOS-GFP-negative MMECs.

#### MMEC conditioned mediae

NOS-GFP-positive and eNOS-GFP-negative MMECs were separately seeded into 6 well-tissue culture plates at a density of 3×10^6^ cells/well. The cells were incubated for 12 hours then washed three times with PBS prior to fresh media being added to the cells. The supernatant was collected 24 hours later and is referred to as eNOS-GFP-positive and eNOS-GFP-negative media, respectively.

#### TNF-α treated podocyte cell culture

Podocytes were seeded in 6 well-plates at a density of 1×10^6^ cells per well and cultured initially at 33°C (propagating condition) prior being cultured at 37°C (differentiating condition). Five days after differentiation had commenced, conditioned media was added to the cells. The medium was changed to 0.1% FBS on day 7. Podocytes were stimulated with 10 ng/ml TNF- α for 36 hours before harvesting.

### Histological assessment

A coronal slice of kidney tissue was fixed in 4% paraformaldehyde and embedded in paraffin. Tissue was cut at 4 μm and stained with hematoxylin, PAS, and Masson's trichrome. The degree of glomerulosclerosis and interstitial fibrosis were measured using Image J software (http://rsb.info.nih.gov/ij/). The percentage of glomerulosclerosis was calculated by dividing the total area of PAS positive staining in the glomerulus by the total area of the glomerulus. Interstitial fibrosis was quantified by dividing the area of trichrome stained interstitium by the total cortical area. The mean value of 20 randomly selected glomeruli or five cortical fields was determined for each section. Five sections were selected from each kidney.

### Antigen Retrieval

Paraffin tissue sections (4 μm) were incubated at 60°C overnight before dewaxing with 2 changes of xylene and 100% ethanol. Tissue sections were immersed in sodium citrate buffer (10 mM sodium citrate, pH 6.0) and heated up in a pressurized cooker to 100°C for 10 minutes. Tissue sections were cooled down to room temperature and prepared for standard immunofluorescence staining procedure.

### Confocal Microscopy

Renal sections were blocked with PBS containing 1% BSA and incubated with rabbit anti-synaptopodin (1∶800) (Sysy antibody, Germany) or rat anti-CD31 (1∶100) overnight at 4°C. Sections were probed with goat anti-rabbit or goat anti-rat with Alexa Fluor 555 conjugate (1∶2000; Molecular Probes, Eugene, OR). Sections were counterstained with 4, 6-diamidino-2 phenylindole (DAPI) to visualize nuclei and mounted with Fluorescence Mounting Medium (Dako Cytomation). Sections were analyzed with an Olympus Fluoview 1000 confocal microscope (Olympus, Tokyo, Japan), FV10-ASW software (version 1.3c; Olympus), oil UPLFL 60x objective (NA1.25; Olympus) at x2 or x3 digital zoom. Contrast and brightness of the images were adjusted further in ImageJ.

### TUNEL Assay

Apoptotic assays were performed by TdT mediated X-dUTP nicked labeling (TUNEL) reaction using ApopTag® Fluorescein In Situ Apoptosis Detection Kit (Merck Millipore, Kilsyth, Vic, Australia). Apoptotic endothelial cells and podocytes were identified by double labelling using TUNEL and anti-CD31 or anti-synaptopodin. Goat anti-rat Alexa Fluor 555 conjugate (1∶2000) and goat anti-rabbit Alexa Fluor 555 conjugate (1∶2000) were used. Sections were counterstained with DAPI.

### Western blotting

Kidney tissues and cell culture samples were sonicated and lysed in 0.4 ml RIPA lysis buffer. The tissue and cell extracts were centrifuged at 3000 rpm and 4°C for 30 minutes to remove cell debris. The protein concentrations were measured by modified Lowry protein assay using BSA as a protein standard (DC protein assay kit, Biorad). Proteins were electrophoresed through a 10% SDS-PAGE gel before transferring to a PVDF membrane. After blocking for 30 minutes at 4°C in blocking buffer (5% skim milk powder in PBS with 0.1% Tween 20), the membrane was incubated overnight with rabbit anti-synaptopodin (1∶8000) or rabbit anti-eNOS (1∶4000) (Santa Cruz Biotechnology, Inc). The membrane was washed and incubated for 30 minutes at room temperature with a goat anti-rabbit antibody conjugated with HRP. After further washing, the membrane was detected with ECL kit (Amersham Pharmacia Biotech, Arlington, IL, USA). α-tubulin and GAPDH were used as internal controls and detected by mouse anti-α-tubulin antibody conjugated with HRP and mouse anti-GAPDH antibody conjugated with HRP. Western blotting images were captured by Kodak 4000 mm and density of the bands was quantitated by using ImageJ (http://rsb.info.nih.gov/ij/).

### Statistical Analyses

Data are mean ± SD with statistical analyses performed using one way or two-way ANOVA from GraphPad Prism 5.0 (GraphPad Software, San Diego, CA) and post test Tukey analysis when appropriate. P<0.05 was considered statistically significant.

## Results

### Characteristics of ADR-Induced Nephropathy in C57BL/6 mice with eNOS deficiency

**Figure 1:**
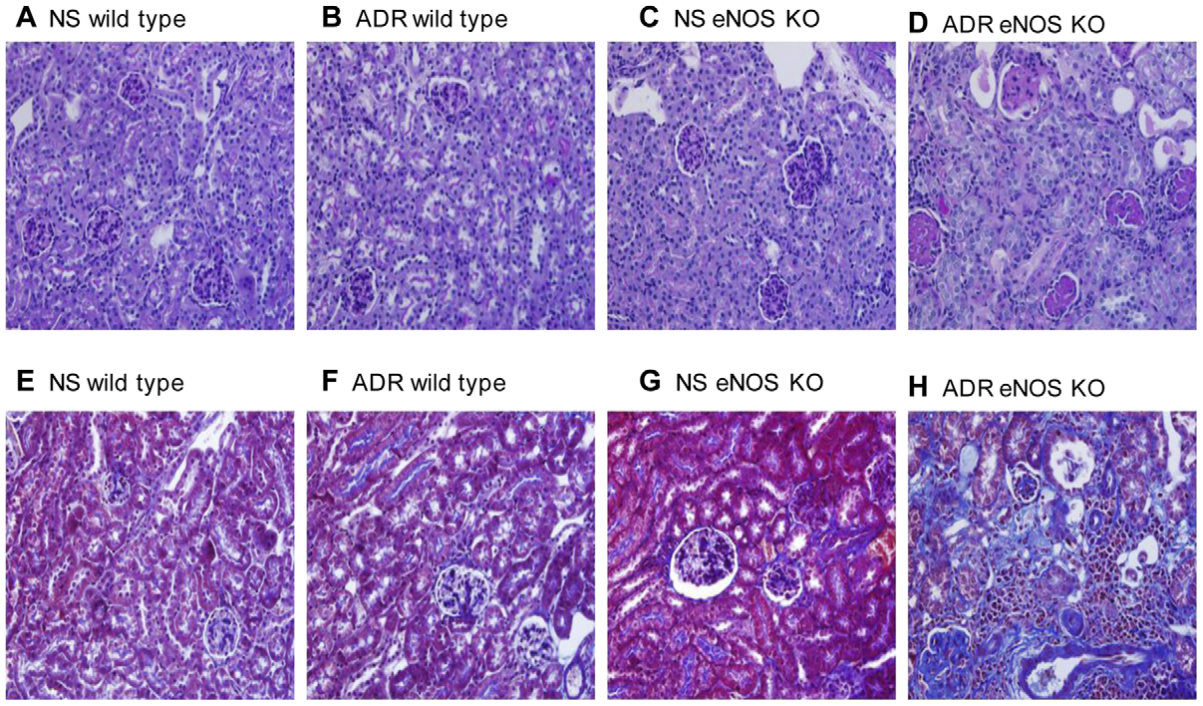
Pathological characterization of ADR-induced nephropathy in C57BL/6 mice with eNOS deficiency. PAS staining of sections from NS (A&C) and ADR-injected (B&D) wild type (A&B) and eNOS-deficient (C&D) mice at day 28. Masson trichrome staining of sections from NS (E&G) and ADR-injected (F&H) wild type (E&F) and eNOS-deficient (G&H) mice at day 28. eNOS-deficient mice with ADR-induced nephropathy exhibited well developed exudative (fibrin-cap) lesions, glomerular sclerosis, interstitial fibrosis and inflammation at day 28. Original magnifications, 400 X.

**Figure 2:**
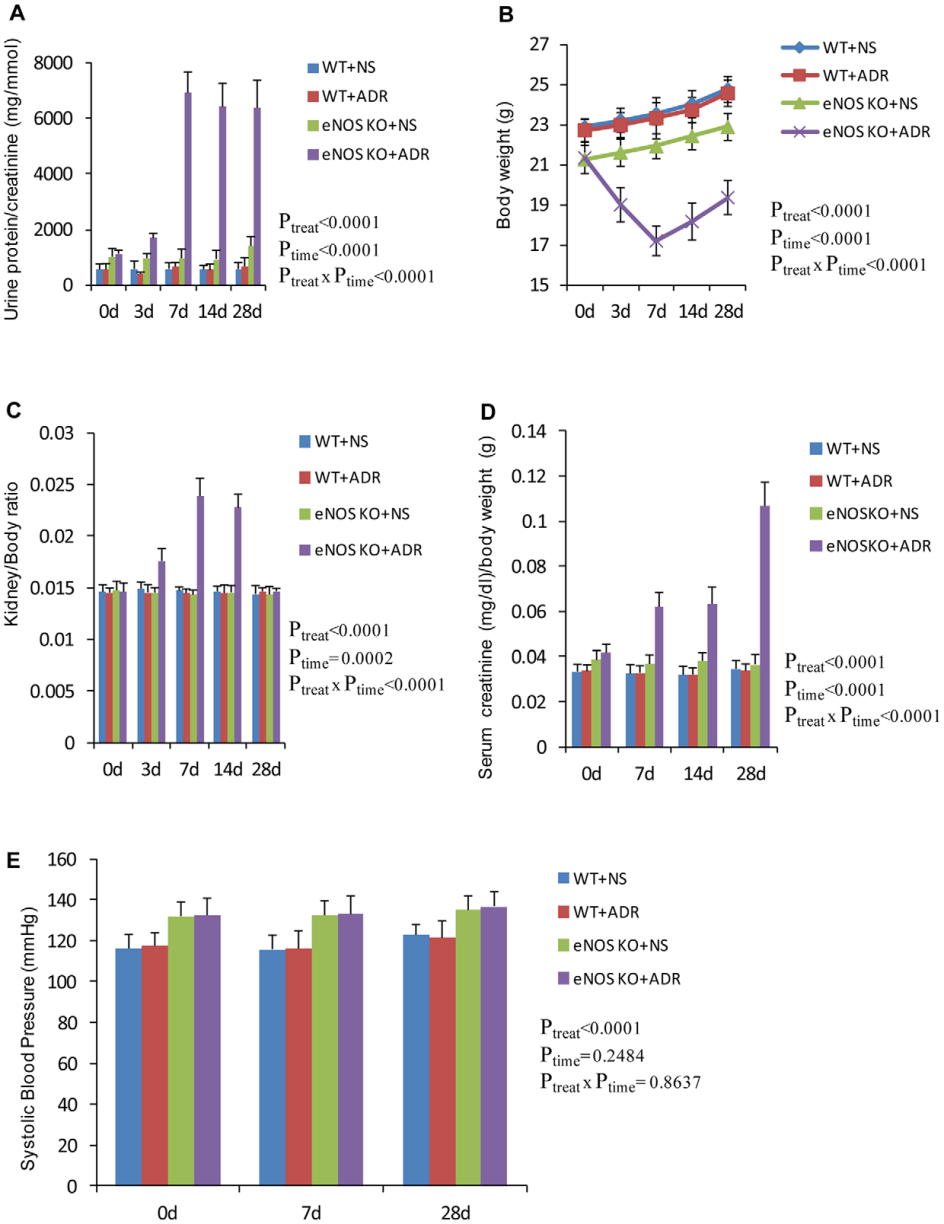
Functional characterization of ADR-induced nephropathy in C57BL/6 mice with eNOS deficiency. **A:** Ratio of urinary protein/creatinine; **B:** Body weight; **C:** Ratio of kidney /body weight; **D:** Serum creatinine and **E:** Systolic blood pressure in NS- and ADR-injected mice. *Two-way ANOVA*; n = 5, data are means ± SD.

**Figure 3:**
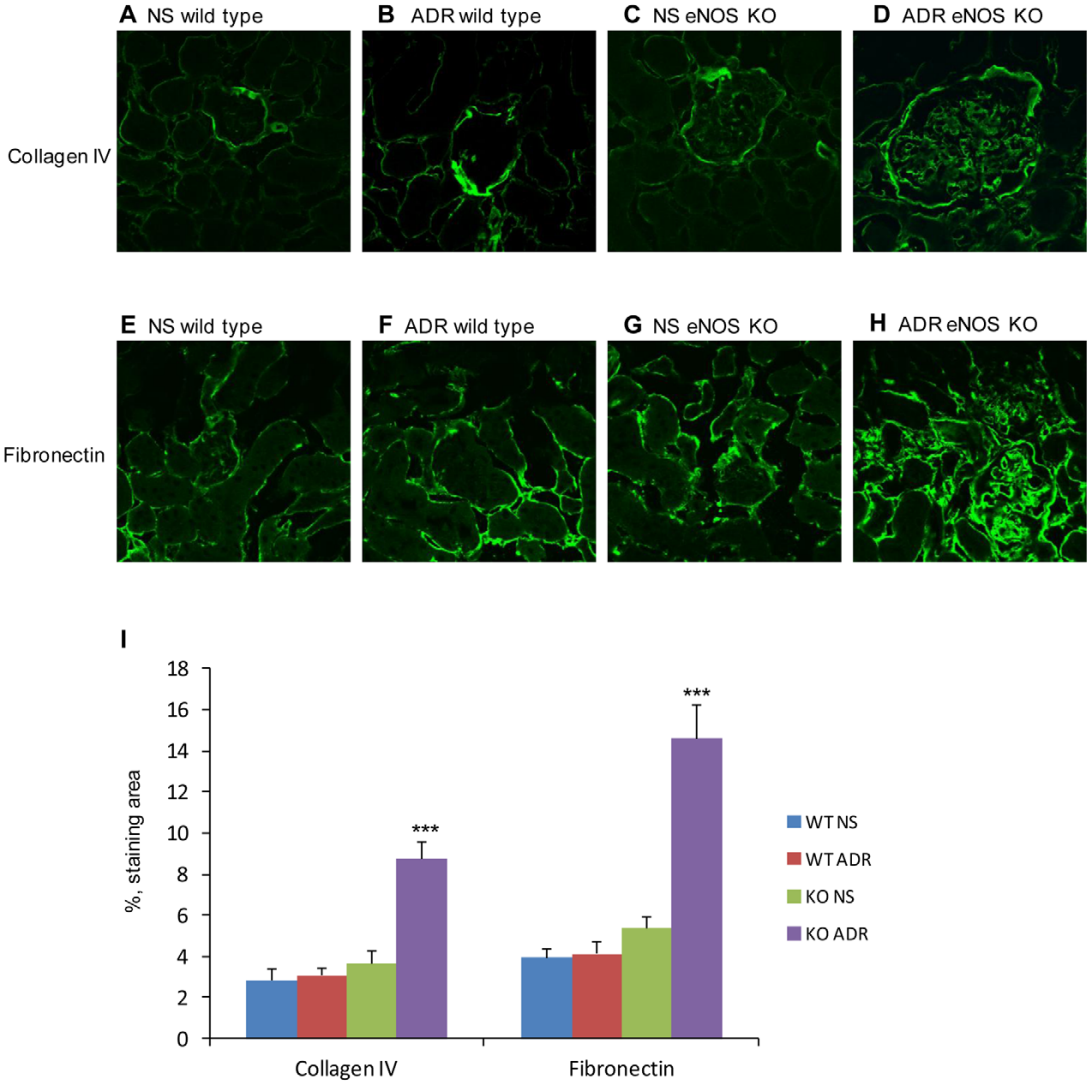
Extracellular matrix products in ADR-induced nephropathy in C57BL/6 mice with eNOS deficiency. Collagen IV (A–D) and fibronectin (E–H) staining sections from NS- (A, C, E & G) and ADR-injected (B, D, F & H) wild type (A, B, E & F) and eNOS-deficient (C, D, G & H) kidneys at day 28. Graph showing quantification of the area of staining for collagen IV and fibronectin. *One-way ANOVA*, n = 5, data are means ± SD. ***: *vs* WT NS, WT ADR and eNOS KO NS, *P*<0.001.

In normal saline (NS)-treated wild type and eNOS-deficient C57BL/6 groups, glomeruli and tubulointerstitium histology were normal (Fig. 1A&C, E&G). A single dose of ADR administration at 10.5 mg/kg body weight in wild type C57BL/6 mice did not induce any significant injury in kidneys (Fig. 1B&F). However, in the ADR-treated eNOS-deficient group, PAS (Fig. 1D) and Masson trichrome staining (Fig. 1H) demonstrated severe histopathological changes including glomerular and tubulointerstitial damage, massive cast formation, glomerulosclerosis, and tubulointerstitial fibrosis. Overt proteinuria appeared 7 days after ADR administration and persisted thereafter (Fig. 2A). In eNOS-deficient mice, the mean body weight decreased quickly after ADR administration and the tendency persisted until day 14, after which body weight recovered gradually (Fig. 2B). Kidney/body ratio in eNOS-deficient mice with ADR treatment increased at day 3, peaked at days 7 and 14 then returned to normal at day 28 (Fig. 2C). Serum creatinine continuously increased following ADR injection in eNOS-deficient mice and peaked at 4 weeks, the experimental end-point (Fig. 2D). In eNOS-deficient mice, high blood pressure persisted during the whole study but there was no significant change in blood pressure between NS-treated and ADR-treated groups (Fig. 2E). Immunostaining demonstrated that the production of collagen IV (Fig. 3 A to D & I) and fibronectin (Fig. 3E to H & I) was significantly increased in ADR-treated eNOS-deficient kidneys compared with NS-treated eNOS-deficient, NS-treated wild type and ADR-treated wild type kidneys. These results demonstrated that ADR administration in eNOS-deficient C57BL/6 mice leads to progressive renal fibrosis that by 4 weeks resembles chronic renal failure with marked functional impairment and severe histopathological alterations. These results suggest that endothelial dysfunction may lead to the development and progression of chronic kidney disease.

### Glomerular endothelial cell injury precedes that of podocytes after ADR administration in eNOS-deficient C57BL/6 mice

**Figure 4:**
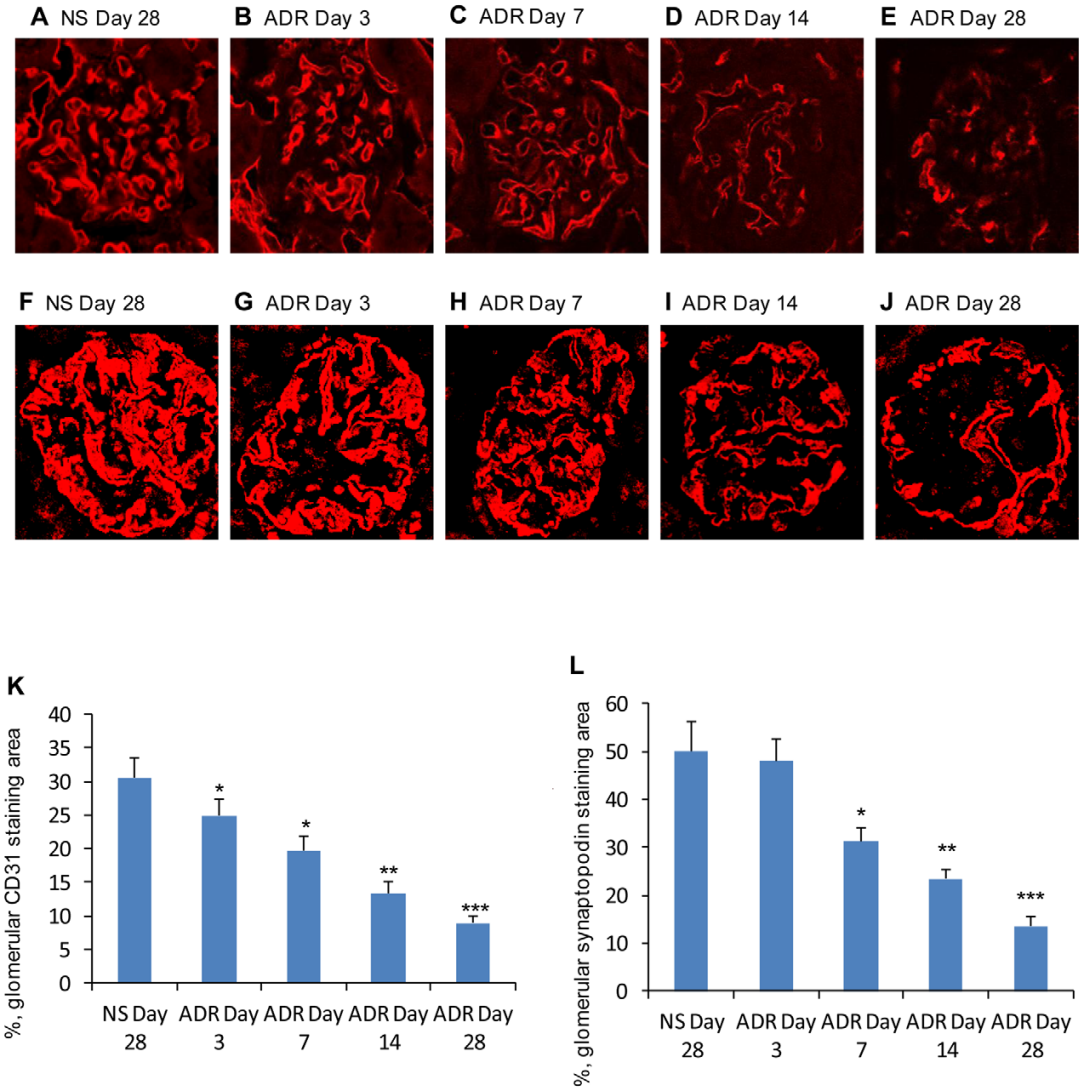
Glomerular endothelial cell and podocyte damage in ADR-induced nephropathy in C57BL/6 mice with eNOS deficiency. Time course of glomerular endothelial cell CD31 (A–E) and podocyte synaptopodin (F–J) staining sections from NS-treated kidneys at day 28 (A&F), ADR-treated kidneys at days 3 (B&G), 7 (C&H), 14 (D&I) and 28 (E&J). Graph showing quantification of the area of CD31(K) and synaptopodin (L) staining. *One-way ANOVA*, n = 5, data are means ± SD. *Vs* NS day 28, * *P*<0.05; **P<0.01; ***P<0.001.

**Figure 5:**
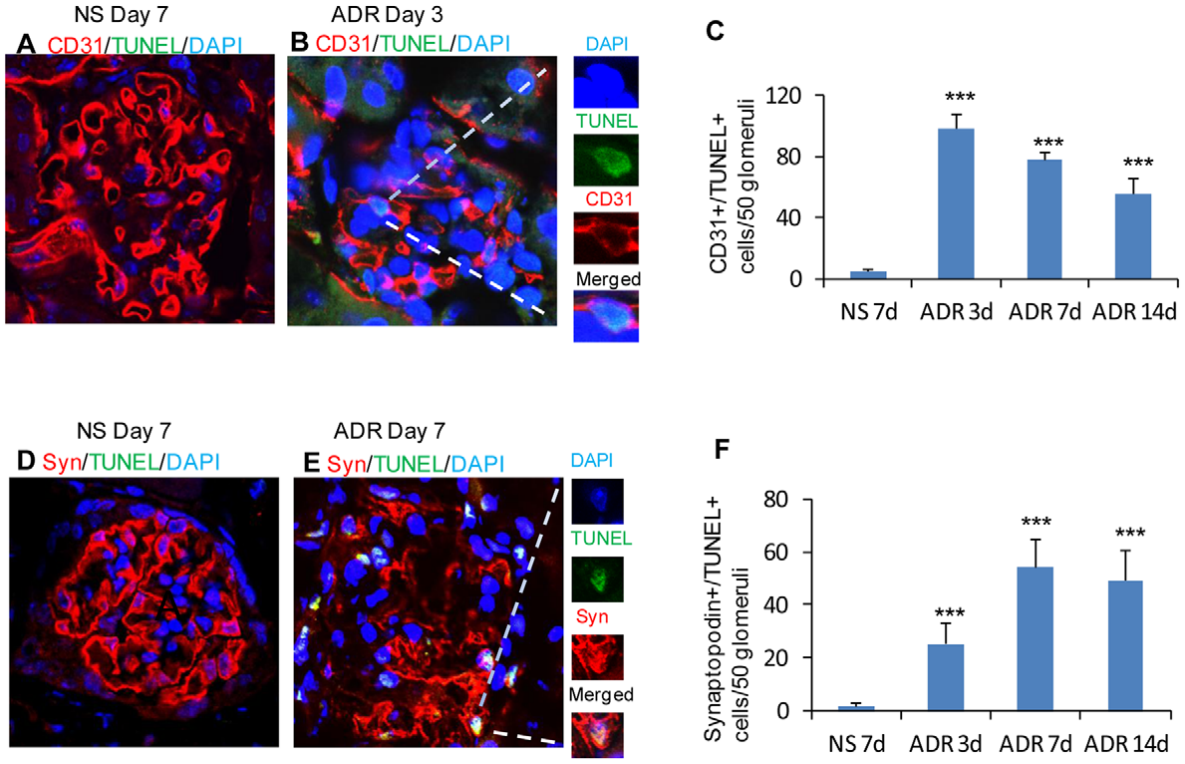
Apoptosis in glomerular endothelial cells and podocytes in ADR-induced nephropathy in C57BL/6 mice with eNOS deficiency. Apoptotic glomerular endothelial cells (A&B) and podocytes (D&E), triple labeled with terminal deoxynucleotidyl transferase-mediated digoxigenin-dNTP nick end-labelling (TUNEL; A, B, D and E, green), anti-CD31 (A&B, red) and anti-synaptopodin (D&E, red), were detected at days 3 (B) and 7 (E) after ADR injection in eNOS-deficient mouse kidneys. Positive apoptotic cells (B&E) were counterstained with DAPI nuclear staining. Sections from NS-treated kidneys (A&D) were used as controls. Quantification of CD31^+^/TUNEL^+^ glomerular endothelial cells (C) and synaptopodin^+^/TUNEL^+^ podocytes in glomeruli (F). Original magnification, 600 X. Magnification in insets, 1200×. *One-way ANOVA*, n = 5, data are means ± SD. ***: *vs* NS day 28, *P*<0.001.

To compare ADR-induced injury in glomerular endothelial cells with that in podocytes in mice with eNOS deficiency, CD31 and synaptopodin staining were performed. The loss of CD31 was evident 3 days after adriamycin administration then persisted until day 28 (Fig. 4A to E & K) while the expression of synaptopodin was significantly reduced 7 days after ADR administration (Fig. 4F to J & L), suggesting that glomerular endothelial cells with eNOS deficiency are more susceptible to injury than podocytes and that endothelial dysfunction plays a critical role in the development and progression of ADR-induced nephropathy. To quantify the rate of apoptosis in glomerular endothelial cells and podocytes, TUNEL was performed in conjunction with CD31 and synaptopodin staining. Positive cells in 50 glomeruli of at least five animals of each group were counted. As expected, the number of glomerular endothelial cells undergoing apoptosis (CD31+/TUNEL+) peaked at 3 days after adriamycin was administered, then gradually decreased at days 7 and 14 (Fig. 5A to C). However, the number of podocytes undergoing apoptosis peaked at 7 days after adriamycin treatment (Fig. 5D to F), demonstrating that adriamycin-induced glomerular endothelial cell injury precedes that of podocytes in eNOS-deficient mice, suggesting that endothelial dysfunction may result in podocyte injury.

### Glomerular endothelial dysfunction precedes podocyte injury in ADR-induced kidney damage in Balb/c mice

**Figure 6:**
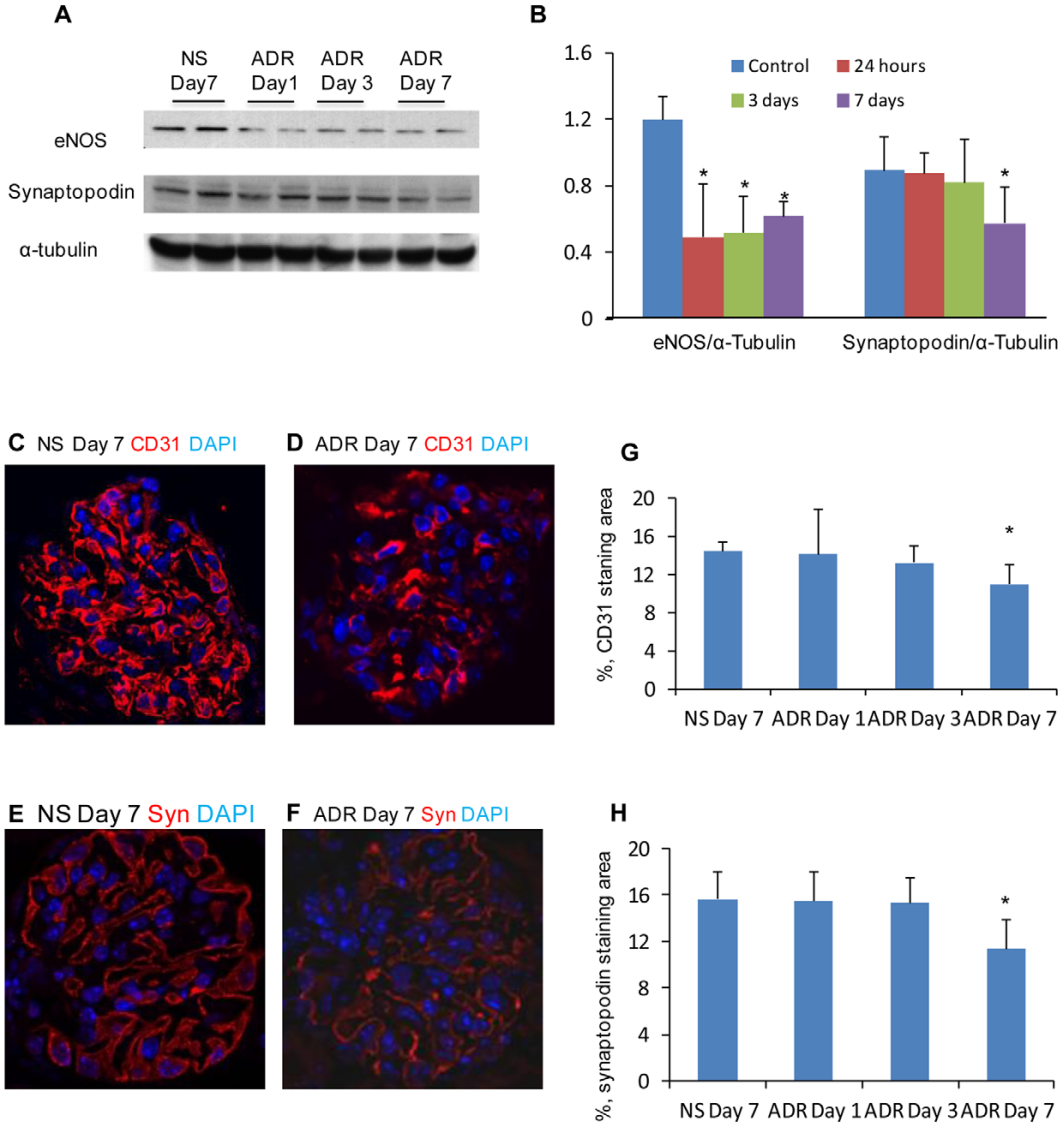
Glomerular endothelial cell and podocyte injury in ADR-induced nephropathy in Balb/c mice. (A) Western blotting detected expression of CD31 and synaptopodin in NS-treated and ADR-treated kidneys in Balb/c mice. (B) Quantification of CD31/α-Tubulin and synaptopodin/α-Tubulin in Western blotting. Immunostaining of CD31+ (glomerular endothelial cells) (D) and synaptopodin+ (podocytes) (F) in ADR-induced nephropathy. NS-treated kidneys were used as normal controls (C&E). Quantification of CD31 (G) and synaptopodin (H) staining in NS-treated and ADR-treated kidneys. *One-way ANOVA*, n = 6, data are means ± SD. *P<0.05.

**Figure 7:**
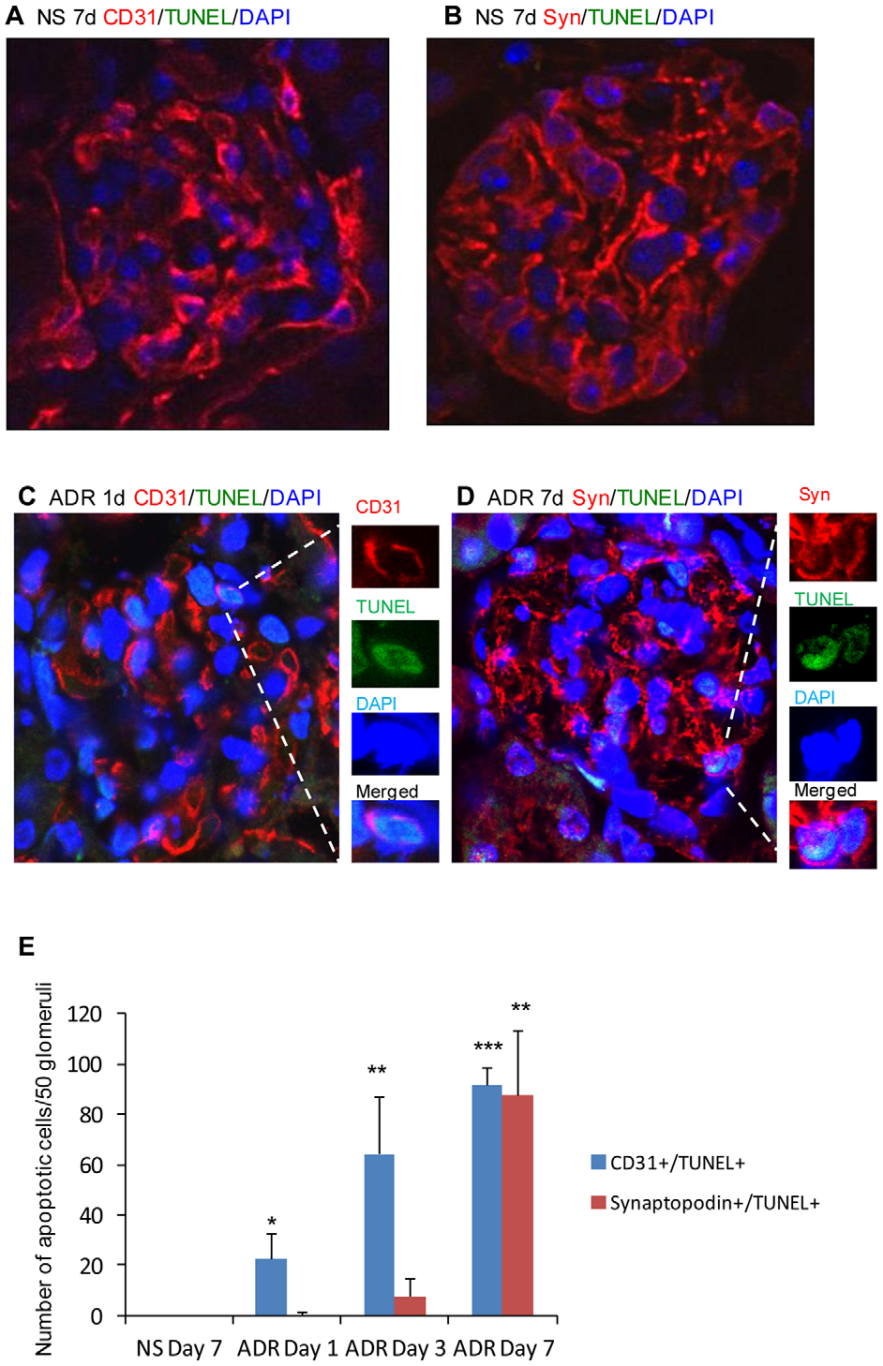
Apoptotic glomerular endothelial cells and podocytes in ADR-induced nephropathy in Balb/c mice. Apoptotic glomerular endothelial cells (A&B) and podocytes (D&E), triple labeled with terminal deoxynucleotidyl transferase-mediated digoxigenin-dNTP nick end-labeling (TUNEL; A, B, D and E, green), anti-CD31 (A&B, red) and anti-synaptopodin (D&E, red), were detected at days 1 (B) and 7 (D) after ADR injection in Balb/c mouse kidneys. Positive apoptotic cells (B&D) were counterstained with DAPI nuclear staining. Sections from NS-treated kidneys (A&C) were used as controls. Quantification of CD31^+^/TUNEL^+^ glomerular endothelial cells and synaptopodin^+^/TUNEL^+^ podocytes in glomeruli (E). Original magnification, 600 X. Magnification in insets, 1200 X. *One-way ANOVA*, n = 6, data are means ± SD. Vs NS day 7, *P<0.05; **P<0.01; ***P<0.001.

It is believed that ADR-induced nephropathy is initiated by podocyte injury followed by overt proteinuria, glomerulosclerosis, tubulointerstitial fibrosis and inflammation in ADR-susceptible mice [35], [36]. In an attempt to address the role of endothelial dysfunction in the development and progression of ADR-induced podocyte injury, the expression of eNOS and synaptopodin were examined by Western blotting in kidneys from Balb/c mice. Interestingly, the down-regulation of eNOS was significantly earlier than that of synaptopodin being prominent 24 hours and 7 days after ADR administration, respectively (Fig. 6A&B). Confocal microscopy demonstrated that CD31 (Fig. 6C, D & G) and synaptopodin (Fig. 6E, F & H) were significantly decreased 7 days after ADR treatment. TUNEL demonstrated that glomerular endothelial cells (CD31+/TUNEL+) and podocytes (synaptopodin+/TUNEL+) undergoing apoptosis could be detected as early as 24 hours in glomerular endothelial cells (Fig. 7C & E) but at 7 days in podocytes (Fig. 7D & E) after ADR treatment compared with NS treatment. This suggests that glomerular endothelial dysfunction and damage precede podocyte injury in an ADR-susceptible mouse strain.

### eNOS overexpression in endothelial cells protects podocytes from TNF-α-induced injury

**Figure 8:**
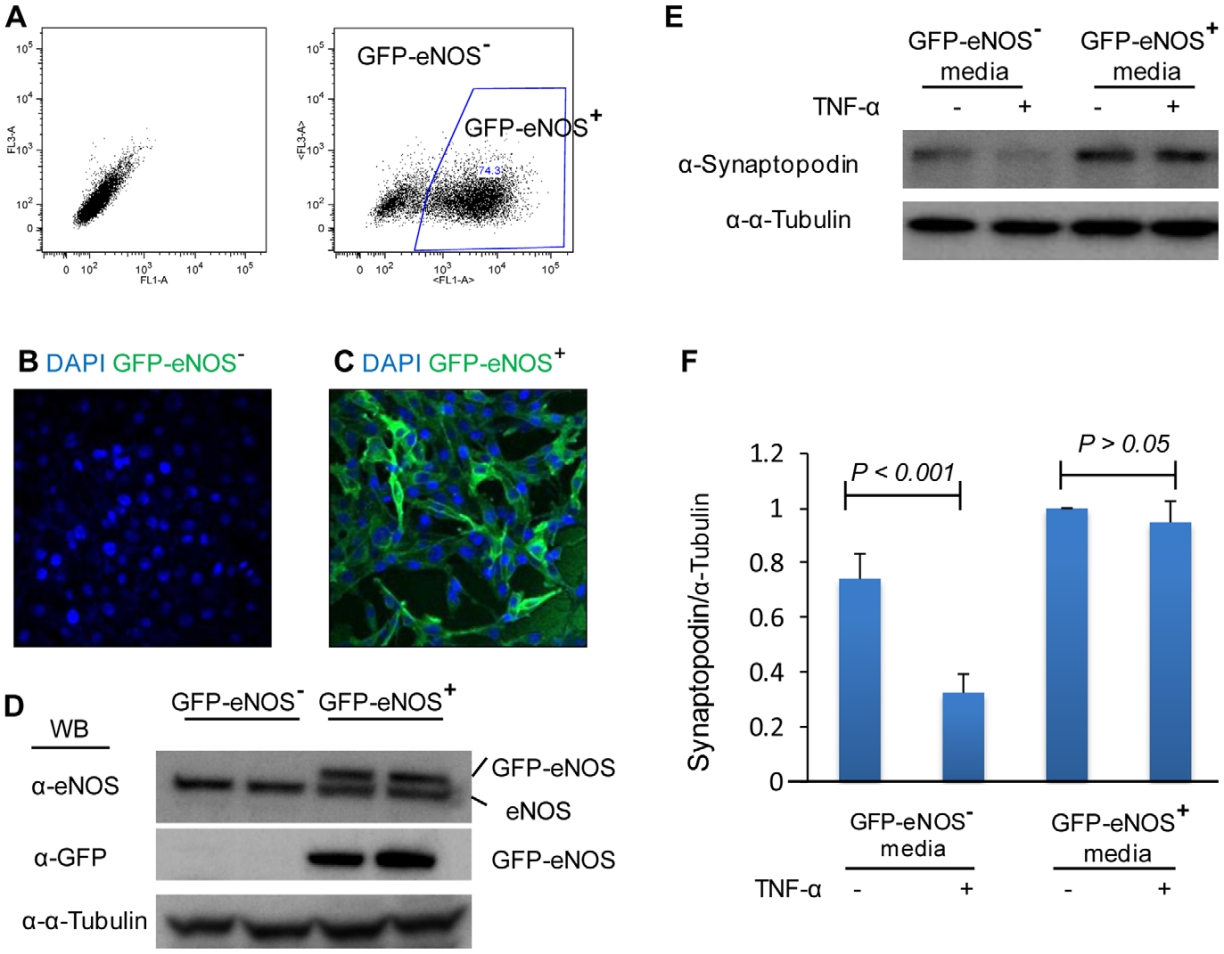
eNOS overexpression protecting podocytes from TNF-α-induced loss of synaptopodin. GFP eNOS – positive (GFP-eNOS^+^) and GFP-eNOS – negative (GFP-eNOS^−^) MMECs were obtained by FACS (A). Confocal microscopy of GFP in GFP-eNOS^−^ (B) and GFP-eNOS^+^ (C) MMECs. (D) Western blotting using anti-eNOS and anti-GFP antibodies to detect endogenous eNOS and overexpression of GFP-eNOS in GFP-eNOS^−^ and GFP-eNOS^+^ MMECs. (E) Conditioned media from GFP-eNOS^−^ and GFP-eNOS^+^ MMECs was added to podocytes in the presence or absence of TNF-α, western blotting demonstrated expression levels of synaptopodin 36 hours after TNF-α stimulation. (F) Quantification of expression levels of synaptopodin by western blotting. *One-way ANOVA*, data are means ± SD.

To further investigate the role of glomerular endothelial cells in the development and progression of podocyte injury, mouse microvascular endothelial cells (MMECs) over-expressing GFP-tagged eNOS were generated. MMECs expressing GFP-tagged eNOS (GFP-eNOS**^+^**) were selected by FACS while GFP-eNOS**^−^**MMECs were used as a negative control (Fig. 8A). Confocal microscopy demonstrated that the majority of the cultured GFP-eNOS^+^ MMECs expressed GFP-tagged eNOS (Fig. 8C) compared with GFP-eNOS**^−^**MMECs (Fig. 8B). Western blotting also confirmed the expression of GFP-eNOS and endogenous eNOS in MMECs (Fig. 8D). Conditioned medium from GFP-eNOS**^+^** MMECs and GFP-eNOS**^−^**MMECs were added to podocytes in the presence or absence of TNF- α. Western blotting demonstrated that TNF-α significantly induced loss of synaptopodin in podocytes under conditioned medium from GFP-eNOS**^−^**MMECs while conditioned medium from GFP-eNOS**^+^** MMECs protected podocytes from TNF-α-induced loss of synaptopodin (Fig. 8E&F), suggesting that eNOS over expression in MMECs may protect podocyte from inflammatory insult.

## Discussion

In the present study using two mouse strains C57BL/6, an ADR resistant strain, and Balb/c, an ADR-susceptible strain, we have demonstrated that one of the important factors in driving ADR-induced nephropathy is the level of expression of eNOS. eNOS deficient C57BL/6 mice when treated with ADR developed overt proteinuria, persistent glomerular endothelial cell and podocyte injury, progressive glomerulosclerosis, tubulointerstitial fibrosis and inflammation. These results suggest that endothelial dysfunction may play a critical role in the development and progression of chronic kidney disease. We also demonstrated that glomerular endothelial cell injury precedes that of podocytes after ADR treatment in both ADR-resistant and ADR-susceptible strains. Using a reciprocal approach we demonstrated that conditioned medium from MMECs over expressing eNOS protected podocytes from TNF-α-induced injury, suggesting that glomerular endothelial cells may also play a protective role in the pathogenesis of chronic kidney disease.

Adriamycin, a putative podocyte toxin [37], induces rapid production of reactive oxygen species and advanced glycation end-products (AGEs) and upregulation of Receptor for AGEs (RAGE) [38]. Guo et al [38] demonstrated that RAGE-deficient mice were protected from ADR-induced podocyte injury, albuminuria and glomerulosclerosis, suggesting that ADR-induced nephropathy is initiated at least partially through RAGE. However, they did not show whether ADR also induced glomerular endothelial cell injury as RAGE is expressed in both podocytes [39] and glomerular endothelial cells [40] though at low levels. Pathological insults, such as ADR treatment [34] and diabetes [40] can significantly increase RAGE expression in both podocytes and glomerular endothelial cells. The interaction of AGEs and RAGE can significantly reduce eNOS mRNA and protein expression in human umbilical vein cords endothelial cells [41]. The present study demonstrated that eNOS deficiency makes C57BL/6 mice, a strain resistant to ADR, susceptible to ADR-induced nephropathy. In Balb/c mice, a susceptible strain, the reduction of eNOS and glomerular endothelial dysfunction appeared as early as 24 hours after ADR treatment, suggesting that both podocyte and glomerular endothelial cell injury contributes to the development and progression of glomerulopathy.

In this study we used a low dose of ADR (10.5 mg/kg). Using a high dose of ADR (25 mg/kg) in C57BL/6 mice Jeansson et al [42] demonstrated a 80% reduction in the thickness of the glomerular endothelial surface layer and significant loss of charge density and size selectivity of the glomerular barrier. They did not show long-term pathological changes in ADR-treated kidneys. Their study suggests that the glomerular endothelial cells may contribute to the development and progression of proteinuric renal diseases. Our study further demonstrated that glomerular endothelial cell dysfunction preceded podocyte injury and that glomerular endothelial cells underwent apoptosis earlier than podocytes, further supporting the notion that besides podocytes, glomerular endothelial cells also play an important role in glomerulopathy.

Earlier studies [43], [44] have shown that mice with eNOS deficiency had significantly elevated blood pressures associated with increase in renin activities. In the present study, ADR treatment did not further alter the increased blood pressures compared with NS treatment in eNOS-deficient mice, suggesting that high blood pressure may contribute to the initiation of ADR-induced kidney injury but ADR-induced kidney damage per se did not have an impact on blood pressure.

Podocytes and glomerular endothelial cells cross-talk through the secretion of cytokines and growth factors [45]–[48]. Sison et al [46] elegantly demonstrated through the use of genetically modified animals that vascular endothelial growth factor-A (VEGF-A) secreted by podocytes binds to VEGFR2 on adjacent endothelial cells to participate in kidney development and to maintain endothelial cell survival and function. Davis et al [47] demonstrated that podocyte-specific expression of angiopoietin-2 induced apoptosis of the glomerular endothelial cells and proteinuria but the podocytes and the GBM remained intact. Slater et al [48] demonstrated that co-culture of human glomerular endothelial cell under laminar shear stress with podocytes resulted in an increase in phosphorylation of Vasodilator-stimulated phosphoprotein at S157 and S239 in podocytes and a decrease in podocyte barrier resistance. These results suggest that glomerular endothelial cells under stress may release mediators to cross-talk with podocytes thus influencing podocyte behaviour. In our study podocytes cultured with conditioned media from MMECs over-expressing eNOS were resistant to TNF-α-induced loss of synaptopodin, providing direct evidence that glomerular endothelial cells may protect podocytes from inflammatory insult through secreting mediators or change their production of a variety of cytokines, proteoglycans and growth factors. What mediators are released from endothelial cells and the exact mechanisms on how endothelial cells influence podocytes requires further investigation.

The other cell type that needs to be considered in the glomerulus is the mesangial cell which expresses nitric oxide (NO) receptors [49]. Mesangial cells require NO to survive and regulate their function [50], [51]. In fact, eNOS deficiency also has an impact on mesangial cells, as evidenced by mesangiolysis [6]–[8]. The interaction between glomerular endothelial cells and mesangial cells warrants further investigation.

In conclusion, our study demonstrated that endothelial dysfunction and damage precedes podocyte injury in ADR-induced nephropathy. In addition, glomerular endothelial cells may protect podocytes through secreting mediators. Understanding the role of glomerular endothelial dysfunction in the pathogenesis of glomerular injury and sclerosis will greatly aid in the design of novel therapeutic approaches for slowing the progressive of renal disease.
